# Statistical Significance Testing for Mixed Priors: A Combined Bayesian and Frequentist Analysis

**DOI:** 10.3390/e24101328

**Published:** 2022-09-21

**Authors:** Jakob Robnik, Uroš Seljak

**Affiliations:** 1 Physics Department, University of California at Berkeley, Berkeley, CA 94720, USA; 2 Lawrence Berkeley National Laboratory, Berkeley, CA 94720, USA

**Keywords:** hypothesis testing, Bayesian statistics, frequentist statistics, statistical mechanics, look-elsewhere effect, exoplanet transit search

## Abstract

In many hypothesis testing applications, we have mixed priors, with well-motivated informative priors for some parameters but not for others. The Bayesian methodology uses the Bayes factor and is helpful for the informative priors, as it incorporates Occam’s razor via the multiplicity or trials factor in the look-elsewhere effect. However, if the prior is not known completely, the frequentist hypothesis test via the false-positive rate is a better approach, as it is less sensitive to the prior choice. We argue that when only partial prior information is available, it is best to combine the two methodologies by using the Bayes factor as a test statistic in the frequentist analysis. We show that the standard frequentist maximum likelihood-ratio test statistic corresponds to the Bayes factor with a non-informative Jeffrey’s prior. We also show that mixed priors increase the statistical power in frequentist analyses over the maximum likelihood test statistic. We develop an analytic formalism that does not require expensive simulations and generalize Wilks’ theorem beyond its usual regime of validity. In specific limits, the formalism reproduces existing expressions, such as the *p*-value of linear models and periodograms. We apply the formalism to an example of exoplanet transits, where multiplicity can be more than $10^{7}$. We show that our analytic expressions reproduce the *p*-values derived from numerical simulations. We offer an interpretation of our formalism based on the statistical mechanics. We introduce the counting of states in a continuous parameter space using the uncertainty volume as the quantum of the state. We show that both the *p*-value and Bayes factor can be expressed as an energy versus entropy competition.

## 1. Introduction

The nature of scientific discovery typically proceeds via the falsification of the null hypothesis via a test that is guided by an alternative hypothesis we wish to compare to. In many cases, we can write the alternative as an extension of the null hypothesis, such that, for example, there is a parameter of the alternative hypothesis whose value is fixed under the null hypothesis. In these situations, the null hypothesis is well-specified in terms of the prior distributions of its parameters, while we may have little or no idea what the prior distribution of the parameters of the alternative hypothesis is. Often, we are performing the search over many parameters, some with a well-specified prior and some without (e.g., the amplitude of the new effect). In this paper, we are interested in these mixed prior situations.

The Bayesian methodology of hypothesis testing compares the ratio of the marginal likelihoods of the two hypotheses to form a Bayes factor [1]. If the prior distribution for the alternative is known, this is a valid methodology that yields optimal results. If the prior is only partially known, the resulting answer is sensitive to the features of the model that we have little control over. In particular, we can always arbitrarily down-weight the alternative hypothesis in the Bayes factor by choosing a very broad prior for its amplitude parameters. This may lead to rejecting the alternative when using a Bayes factor. This is not justifiable when the prior is poorly known: the end result can be a missed discovery opportunity. The opposite situation can also occur, where the chosen prior and the corresponding Bayes factor are too optimistic for the alternative hypothesis.

To overcome the arbitrariness of the prior choice, one could try to learn the prior from the data under some hyperparametrization, leading to a hierarchical or empirical Bayesian analysis [2,3]. This approach is not possible if we have no data that can inform the hyperparameters, as is the case when testing a new model (such as a new theory or a new phenomenon) that has never been observed before: a test of a new model should therefore not depend on the reported Bayes factor alone.

Various test statistics have been proposed that attempt to remove the dependence of the hypothesis test on the prior. They fall into two categories. One is basing the test on the posterior rather than the prior. One approach is to compare the posterior predictive data densities under both hypotheses, which can also be related to the cross-validation [4]. Other proposed test statistics include suspiciousness [5] (the posterior averaged log-likelihood ratio) and the e-value [6] (the posterior probability of the parameters which have the posterior density under the alternative higher than the posterior density under the null).

A common failure of the posterior-based hypothesis tests is that they do not account for the look-elsewhere effect or the effective multiplicity of the test: if we perform N tests, the probability of having one of them with an anomalous value under the null hypothesis is increased by N.

A related class of methods are the so-called information criterion heuristics. The AIC and WAIC are meant to be approximations to the posterior predictive densities, and as such, they are closely related to the cross-validation [4]. Another is the Bayesian information criterion (BIC), which is meant to approximate the Bayes factor, but the approximations do not hold for the class of scanning parameters we are concerned about in this paper. Thus, the look-elsewhere effect is not correctly taken into account by these ICs either, even if we account for the “effective number” of dimensions [5]. This is because the look-elsewhere effect does not depend only on the dimensionality difference between the null and the alternative but also on the prior range of the additional parameters: if one searches over a broader prior, the multiplicity increases, and the look-elsewhere effect is more severe.

One typical example is searching for a signal in a time series, such as a localized feature (e.g., planet transit in stellar flux): the signal could be anywhere in the time series, and we must pay the multiplicity penalty for looking at many time stamps. The larger the scan, the more false positives our search will produce. This is completely ignored if we only look at the posterior, which is typically very localized.

An alternative approach is that of frequentist hypothesis testing, which is defined in terms of the false-positive rate of the null hypothesis (the *p*-value, or Type I error). This is independent of the validity of the assumed prior for the alternative hypothesis: we can rule out the null hypothesis even in the absence of a well-developed alternative hypothesis. However, to do so, we need a test statistic, and the best test statistic is the one developed based on the expected properties of an alternative hypothesis. In some situations, we may have a known prior distribution for some parameters, and taking the advantage of this information will increase the power of the test statistic.

The strength of the Bayes factor is thus that there are parameters other than the amplitude that have well-specified priors, for which Bayesian hypothesis testing has Occam’s razor built-in [7], and it automatically accounts for effects, such as the look-elsewhere effect [8]. For example, if we scan for a signal with a large template bank, we must account for the trials factor, and the corresponding *p*-value significance is increased [9,10]. There is no single established frequentist procedure to do this, while the Bayes factor automatically accounts for it. In this case, the dependence on the prior in the Bayes factor analysis is an asset. In many realistic situations, the alternative hypothesis has several parameters, some with well-specified priors and some without. This paper aims to address these situations from both the Bayesian and frequentist perspectives and proposes a solution that takes advantage of both methodologies. The main themes of this paper are:

- The Bayes factor is the test statistic with the highest power and should be used even in frequentist analyses, assuming some of the priors are informative (known).
- For many applications, some of the priors are known, and some are unknown. This mixed prior information requires an analysis that combines the Bayes factor with the frequentist *p*-value.
- For mixed prior problems with some unknown priors, the frequentist *p*-value or Type I error (the false-positive rate) evaluated on the Bayes factor is a better way to summarize the significance of the alternative hypothesis than the Bayes factor itself. In many situations, this can be performed analytically without the simulations.

The Neyman–Pearson lemma guarantees that the maximum likelihood ratio (MLR) is the highest power test statistic for a simple alternative hypothesis with no free parameters. Similarly, the Bayes factor is the test statistic with the highest power (it minimizes the Type II error (the false-negative rate) at a fixed Type I error) if the alternative hypothesis has multiple parameters with some prior information [11,12]. This is because the Bayes factor optimally summarizes the prior information we have about the alternative hypothesis. It does not mean that the estimated Type II error is correct, as the prior we assumed could be wrong. It does however mean that we cannot find a better test statistic on a Type II error without knowing a better prior.

The main contributions of this paper are:

- The systematic treatment of the hypothesis testing with only partial prior information.
- Analytic methods for the evaluation of the *p*-value with the Bayes factor test statistic. Our formalism enables evaluating the *p*-value without running expensive simulations and generalizes the Wilks theorem to non-Gaussian posteriors and high *p*-values.
- The interpretation of the results in terms of the statistical mechanics, using concepts such as the counting of states, uncertainty quantification, and entropy. These connections have been explored before in the context of Bayesian statistics [13]; here, we extend it to the frequentist statistics.

The outline of the paper is as follows: In Section 2, we define the Bayesian hypothesis testing. In Section 3, we develop an analytic formalism for computing the false-positive rate. In Section 4, we apply this formalism to practical examples, notably an exoplanet transit search. In Section 5, we offer an interpretation of the developed formalism based on statistical mechanics.

## 2. Bayesian Hypothesis Testing

We are given some data x and want to test them against competing hypotheses. We will assume there is a single null hypothesis $H_{0}$, which assumes there is no discovery in the data, and a collection of the alternative hypotheses $H_{1}$, all predicting some new signal. For example, when we are looking for a planet transit in the time-series data, the null hypothesis predicts that the stellar variability and noise alone are responsible for the flux variations, while the alternative hypothesis also predicts that the presence of the exoplanet transit dips. There are multiple alternative hypotheses because we do not know the planet’s properties, such as its period, phase, or amplitude, so the alternative hypothesis is not simple and has parameters we need to vary.

The Bayesian approach to hypothesis testing is to examine the ratio of the marginal likelihoods, i.e., the Bayes factor

(1) $$B=\frac{p(x|H_{1})}{p(x|H_{0})},$$

where the marginal integral is

(2) $$p\left(x|H_{1}\right)=\int p\left(x|z\right)p\left(z\right)dz.$$

Here, p(z) is the prior for the alternative hypothesis parameters and p(x|z) is the data likelihood under a general z. A typical situation is that the null hypothesis corresponds to some specific values of z, such as $z_{1}=0$, where $z_{1}$ is the amplitude of the signal for the alternative hypothesis.

We are interested in the posterior odds between the competing hypotheses which follow directly from the Bayes factor by $B\;P\left(H_{1}\right)/P\left(H_{0}\right)$, where $P(H_{0})$ and $P(H_{1})$ are the prior probabilities. We typically assume the prior probabilities of the two hypotheses to be equal, in which case the Bayes factor is also the posterior odds, and in the following, we will focus on the Bayes factor only.

The Bayes factor is an integral over all possible alternative hypotheses, but usually, a relatively small range of parameters, where the data likelihood peaks, dominates the integral. In this case it suffices to apply a local integration at the peak of the posterior mass, which is often (but not always) at the maximum a posteriori (MAP) peak, where the posterior density peaks. If the location of the highest posterior mass peak under $H_{1}$ is $\hat{z}$, this gives the Bayes factor as [8]

(3) $$B=\frac{p(x|\hat{z})}{p(x|H_{0})}\;p\left(\hat{z}\right)V_{\mathrm{post}}\left(x\right).$$

The Bayes factor depends on the MLR at the peak $\exp\left(E\right)\equiv p\left(x|\hat{z}\right)/p\left(x|H_{0}\right)$, alternative hypothesis prior $p(\hat{z})$, and the posterior volume $V_{\mathrm{post}}\left(x\right)$, which is a result of the local integration of the posterior ratio $p\left(z|x\right)/p\left(\hat{z}|x\right)$ around the peak. It can be approximated using the Laplace approximation as $V_{\mathrm{post}}\approx {\left(2\pi \right)}^{d/2}\sqrt{\det\Sigma }$, where *d* is the dimension of the parameter space and Σ is the covariance matrix, given by the inverse Hessian of the negative log-likelihood evaluated at $\hat{z}$,

(4) $${\left(\Sigma^{-1}\right)}_{ij}=-\partial_{i}\partial_{j}\ln{\left[p\left(x|z\right)p\left(z\right)\right]}_{z=\hat{z}}.$$

Here, $\partial_{i}$ is the derivative with respect to $z_{i}$. The choice of prior is subjective, and when the prior is known, it is informative and should be used. When the prior is not known, an option for a non-informative prior is Jeffrey’s prior, which is given by the square root of the determinant of the Fisher information matrix. Fisher matrix is the data-averaged Hessian:

(5) $$I_{ij}\left(z\right)=\langle -\partial_{i}\partial_{j}\log p\left(x|z\right)\rangle ,$$

where 〈f(x,z)〉(z)≡∫f(x,z)p(x|z)dx. Note that the Jeffrey’s prior is an inverse of the data-averaged posterior volume, computed under the Laplace approximation. Therefore, it will on average cancel out the parameter dependence of $V_{\mathrm{post}}\left(\hat{z}\right)$ in the Bayes factor, making it dependent only on the local MLR.

We can generalize these concepts to non-Gaussian posteriors. As we will show in Section 4.3, the Laplace approximation can be poor if the posterior is non-Gaussian, but the local Bayes factor integration is still well-defined, in which case Equation (3) can be viewed as the definition of $V_{\mathrm{post}}$ (we use this in, e.g., Equations (A8) and (25)). The properly generalized Jeffrey’s prior is then $p\left(z|H_{1}\right)\propto 1/\langle V_{\mathrm{post}}\left(z\right)\rangle$. This is the prior, which on average makes the Bayes factor directly proportional to the MLR. Such prior choice may be called non-informative, as only the likelihood is used to test the two hypotheses.

## 3. Frequentist Hypothesis Testing

In frequentist hypothesis testing, we first define a test statistic, which should be chosen to maximize the contrast against the alternative hypothesis. As guaranteed by the Neyman–Pearson argument [11,12], the optimal statistic between two well-specified hypotheses are the posterior odds. Any monotonically increasing function of the posterior odds is an equally good test statistic in the frequentist sense, so for convenience, we will work with F=logB.

In frequentist methodology, we quantify a test statistic using its Type I false-positive rate (*p*-value) under the null hypothesis P(F>F(x)). If this number is sufficiently small, the null hypothesis is rejected. Unlike the posterior odds, the *p*-value is independent of the correctness of the prior of the alternative hypothesis: it only depends on the null hypothesis itself. This is a great advantage of the *p*-value over the posterior odds and is the main reason why posterior odds have not been widely adopted for hypothesis testing, even in Bayesian textbooks [4,14].

While the posterior odds can be derived entirely from the data using the likelihood (the likelihood principle), the *p*-value is given by the frequency distribution of the test statistic under the null hypothesis, which generally requires simulating the null hypothesis many times to obtain it. Because this requires evaluating the test statistic for each simulation, and because we argue the test statistic itself should be the posterior odds, this can be significantly more expensive in the frequentist methodology than evaluating posterior odds once on the data, as performed in the Bayesian methodology. For this reason, we will study analytic techniques for evaluating the false-positive rate. The discussion here will not be rigorous, so we rederive the results under the Gaussian likelihood assumption in Appendix A.

The log-Bayes factor *F* and the maximum log-likelihood ratio *E* are functions of a particular data realization, but here we will approximate them solely as a function of the local MAP parameters $\hat{z}$ (for a motivating example, see Appendix A.2). Note that this is not possible in general, for example, if multiple correlated peaks contribute to the Bayes factor or if the data realizations are very discrete. Nevertheless, here we proceed with this assumption and note that the *p*-value of the Bayes factor can then be inferred from the distribution of the parameters $\hat{z}$ under the null hypothesis $p(\hat{z}|H_{0})$. If the prior is correct, the Bayes factor correctly predicts the relative occurrence of the two hypotheses in a long series of trials:

(6) $$B=p\left(x|H_{1}\right)/p\left(x|H_{0}\right)=p\left(\hat{z}|H_{1}\right)/p\left(\hat{z}|H_{0}\right).$$

The distribution $p(\hat{z}|H_{1})$ follows the prior: each sample with local MAP parameters $\hat{z}$ will have a true value of parameters z approximately within $V_{\mathrm{post}}$ of $\hat{z}$. Thus, as long as the prior is sufficiently smooth relative to the posterior, we have $p\left(\hat{z}|H_{1}\right)\approx p\left(\hat{z}\right)$. Therefore, the distribution of the local MAP parameters under the null hypothesis is

(7) $$p\left(\hat{z}|H_{0}\right)=p\left(\hat{z}|H_{1}\right)B{\left(\hat{z}\right)}^{-1}\approx p\left(\hat{z}\right)e^{-F(\hat{z})}.$$

Note that the distribution $p(\hat{z}|H_{0})$ is independent of the prior because the prior dependence of the Bayes factor cancels $p(\hat{z}|H_{1})$. Therefore, Equation (7) holds regardless of the correctness of the prior. The *p*-value is found by integrating over the parameters which yield at least the desired test statistic F(x):

(8) $$\begin{array}{cc} P_{\mathrm{asym}}\left(F>F\left(x\right)\right) & =\int_{F\left(\hat{z}\right)>F\left(x\right)}p\left(\hat{z}\right)e^{-F(\hat{z})}d\hat{z}=\int_{F(x)}^{\infty }p\left(F\right)e^{-F}dF. \end{array}$$

Here, $p\left(F\right)=\int \delta \left(F-F\left(\hat{z}\right)\right)p\left(\hat{z}\right)d\hat{z}$ is the prior, marginalized over the parameters $\hat{z}$ which yield *F*. This result is saying that the probability density of finding some *F* under the null hypothesis is given by the probability density of finding it under the alternative hypothesis prior, but exponentially damped with *F*. Note that Equation (8) is equivalent to

(9) $$P_{\mathrm{asym}}\left(F>F\left(x\right)\right)=\int_{F\left(\hat{z}\right)>F\left(x\right)}\frac{e^{-E(\hat{z})}}{V_{\mathrm{post}}\left(\hat{z}\right)}d\hat{z},$$

so the region of integration depends on the prior, but the integrand does not. This means that the prior selects the parameter range where the false positives can be generated, but the rate at which they are generated at those parameters is prior independent.

These expressions are useful when we have an analytic expression for the posterior volume and can perform the integral analytically (see examples in Section 4.1 and Section 4.2). Here, we will derive an approximation which directly relates the *p*-value to the Bayes factor, so it is applicable even if the Bayes factor is only available numerically.

Assuming that the alternative has the amplitude parameter whose prior is separable from the other parameters $p\left(z\right)=p_{1}\left(z_{1}\right)p_{>1}\left(z_{>1}\right)$ and whose posterior volume can be approximated by the Laplace approximation $\sqrt{2\pi }\sigma_{1}$, we rewrite Equation (3) and define the reduced Bayes factor $B_{>1}\left(x\right)$ as

(10) $$B\left(x\right)=p_{1}\left({\hat{z}}_{1}\right)\sqrt{2\pi }\sigma_{1}\;B_{>1}\left(x\right).$$

Computing $B_{>1}$ is completely analogous to computing the Bayes factor, but there is no need to perform the $z_{1}$ integral as this is performed analytically by the Laplace approximation.

Typically, the variations of $V_{\mathrm{post}}\left({\hat{z}}_{1}\right)$ are much slower than the likelihood suppression $e^{-E({\hat{z}}_{1})}$ so we may evaluate the ${\hat{z}}_{1}$ integral in (9) by considering the posterior volume to be a constant evaluated at the observed local MAP parameters. This gives

(11) $$P_{\mathrm{asym}}\left(F>F\left(x\right)\right)\approx \frac{p_{1}\left({\hat{z}}_{1}\right)}{B\left(x\right)|\frac{dE}{d{\hat{z}}_{1}}|}=\frac{1}{B_{>1}\left(x\right)\sqrt{4\pi E(x)}},$$

implying that the *p*-value can be inferred directly from the observed reduced Bayes factor $B_{>1}$ with no need to perform any additional integrals.

### Non-Asymptotic p-Value

We have found an analytic expression for the false-positive rate; however, it is only valid when the false-positive rate is low. By following [8], we will show that this result can be easily extended to all *p*-values if the posterior volume is much smaller than the prior volume, so precisely when the look-elsewhere effect is important.

Let us partition the alternative hypothesis manifold in *K* smaller manifolds and consider searches over the smaller manifolds. Let us choose the partition in a way that FPR in all the small searches is the same and call it $P_{\mathrm{small}}\left(F>F\left(x\right)\right)$.

Suppose the posterior volume is much smaller than the prior volume. In that case, most data realizations will have their posterior volume well within the prior range of some smaller manifold, even when *K* is relatively large. Therefore, the probability of not finding a false positive in the original search equals the probability of not finding it in any of the small searches:

(12) $$1-P\left(F>F\left(x\right)\right)=\{1-P_{\mathrm{small}}\left(F>F\left(x\right)\right){\}}^{K}.$$

If *K* is large, $P_{\mathrm{small}}\left(F>F\left(x\right)\right)$ becomes small, and we can compute it using the asymptotic expression of Equation (8) or (11): $P_{\mathrm{small}}\left(F>F\left(x\right)\right)=P_{\mathrm{asym}}\left(F>F\left(x\right)\right)/K$. Taking the large *K* limit, we find

(13) $$P\left(F>F\left(x\right)\right)=1-\exp\{-P_{\mathrm{asym}}\left(F>F\left(x\right)\right)\}.$$

This is a continuous parameters generalization of Sidák correction, which itself is a generalization of Bonferroni correction, which is commonly used for discrete states where the trials factor is a well-defined concept and referred to as multiple test comparison.

## 4. Results

In this section, we apply the developed formalism to three examples with increasing complexity. We start with the linear model and periodogram and compare our results with the literature. We then turn to the more realistic analysis of the exoplanet transit search.

In all cases, the data are a real vector $x\in R^{n}$. The null hypothesis assumes the data are independently identically distributed (IID):

(14) $$p\left(x|H_{0}\right)=\prod_{k=1}^{n}q\left(x_{k}\right),$$

where *q* is the probability density function of the noise. The alternative predicts some feature in the data, such that the residuals x−m(z) are described by the null hypothesis. Note that in the exoplanet case, we will need to go to the Fourier basis and normalize by the power spectrum to make the data IID.

### 4.1. Linear Model

In this first example, the noise is standard Gaussian distributed and the model is a linear superposition of *d* features $m_{i}\in R^{n}$:

(15) $$m_{\mathrm{linear}}\left(w\right)=\sum_{i=1}^{d}w_{i}m_{i}.$$

Without the loss of generality, we may assume the features are orthonormal $m_{i}\cdot m_{j}=\delta_{ij}$ by applying the Gram–Schmidt algorithm if they are not.

The MAP model is a projection of the data on the model plane ${\hat{w}}_{i}=x\cdot m_{i}$ and has the log-MLR $E=\frac{1}{2}\sum_{i=1}^{d}{\hat{w}}_{i}^{2}$. The posterior is Gaussian, so the Laplace approximation is exact and gives the posterior volume ${\left(2\pi \right)}^{d/2}$.

Often, we do not have any prior information, and it makes sense to adopt the Jeffrey’s prior, which is uniform for the linear parameters. For simplicity, we assume the prior volume to be a ball with $\sum_{i=1}^{d}w_{i}^{2}<w_{max}^{2}$. The Bayes factor (3) is

(16) $$B=\frac{{\left(2\pi \right)}^{d/2}e^{E}}{V\left(B^{d}\right)w_{max}^{d}},$$

where $V(B^{d})$ is the volume of the *d*-dimensional unit ball. Note that the Bayes factor cannot be taken at a face value because it is sensitive to the unknown amplitude cutoff $w_{max}$. Furthermore, there is no advantage in using the Bayes factor over the MLR because *F* and *E* only differ by a constant and are therefore equally good as frequentist test statistics. We will therefore use *E* as a test statistic in this example. The posterior volume is a constant, so the integral of Equation (9) just picks up the volume of the constant-likelihood surface, which is a d−1-dimensional sphere. We obtain

(17) $$P\left(E>E\left(x\right)\right)=\int_{E(x)}^{\infty }\frac{E^{d/2-1}\;e^{-E}}{\Gamma (d/2)}dE,$$

where we have used that the volume of a d−1-dimensional sphere is $2\pi^{d/2}/\Gamma \left(d/2\right)$, with Γ the Gamma function. Note that the *p*-value is independent of the unknown amplitude cutoff $w_{max}$.

The resulting cumulative distribution function is a $\chi^{2}$-distribution with *d* degrees of freedom, and we reproduce the well-known result that $\chi^{2}$ of a linear model with *d* features is distributed as a $\chi^{2}$ distribution with *d* degrees of freedom [15]. The *p*-value is increasing with *d* at a constant $\chi^{2}=2E$, which is a reflection of the entropy versus energy competition discussed in Section 5: there are more states on the shell of a constant-energy *E* if *d* is higher.

### 4.2. Periodogram

In this example, we are given *n* time-series measurements $x_{i}=x\left(t_{i}\right)$ with Gaussian uncertainties σ. We are searching for harmonic periodic signals [16,17,18]:

(18) $$m_{i}\left(z\right)=z_{1}\sigma \sqrt{2/n}\;\sin\left(\omega t_{i}+\phi \right).$$

Here, $z_{1}$ is the signal’s amplitude, ω is the unknown frequency, and ϕ is the phase. We have introduced an additional normalizing factor $\sigma \sqrt{2/n}$ for later convenience. We assume a uniform prior on all three parameters, so

(19) $$p\left(z_{1},\omega ,\phi \right)=\frac{1}{z_{max}\;\omega_{max}\;2\pi },$$

where $z_{max}$ is some arbitrary large cutoff on the amplitude and $\omega_{\max}$ is set by some physical properties of the signal or by the experimental limitations, e.g., by the Nyquist frequency.

Using Equations (A7) and (A15), we compute the Bayes factor

(20) $$B=\frac{\sqrt{6\pi }\;e^{E}}{z_{max}\;\omega_{max}T\;E},$$

which again suffers from the unknown amplitude cutoff, so we turn to the frequentist analysis to interpret the Bayes factor. We observe that the Bayes factor and the MLR are a simple function of one another, so their null distributions are related by the change in variable formula $p\left(F|H_{0}\right)=|dF/dE|\;p\left(E|H_{0}\right)=\left|1-1/E\right|\;p\left(E|H_{0}\right)$.

Once again, the posterior volume is independent of the $z_{>1}$ parameters, and the integral (9) picks up the volume of the constant-likelihood surface, which is, in this case, a cylinder, so $2\pi \omega_{max}$:

(21) $$P\left(E|H_{0}\right)=\int_{E(x)}^{\infty }\frac{2\pi \omega_{max}}{B2\pi \omega_{max}z_{max}\frac{dE}{d{\hat{z}}_{1}}}dE=\frac{T\omega_{max}}{\sqrt{12\pi }}\int_{E(x)}^{\infty }E^{1/2}e^{-E}dE,$$

where $E=\frac{1}{2}{\hat{z}}_{1}^{2}$ due to the convenient normalization of the template.

Note that while the amplitude cutoff cancels in the *p*-value, the frequency cutoff does not. This is the look-elsewhere effect: the larger the frequency space search, the larger the false-positive rate at a fixed MLR.

These results agree with the expressions of [19], which use the formalism of [20,21]. This mathematical formalism is based on the extremes of random processes of various distributions, such as the gamma ($\chi^{2}$) distribution, and needs to be derived separately for each distribution. In addition, this formalism formally only gives an analytic lower limit to the corresponding extreme value distributions, while in practice, equality is assumed without proper justification. Our formalism provides a different derivation that results in the same expressions in the periodogram case. However, here, we do not consider the more challenging periodogram problem with sparse data sampling and correlated noise.

### 4.3. Exoplanet Search

As a more complex non-trivial example of the formalism we developed, we consider exoplanet detections in the transit data, where the planet orbiting the star dims the star when it transits across its surface. We have a time series of a star’s flux measurements $x_{i}=x\left(t_{i}\right)$. In the absence of a transiting planet, the data are described by a stationary correlated Gaussian noise modeling the stellar variability. Note that the long-term trends in the Kepler data are removed by the preprocessing module [22] and outliers can be efficiently Gaussianized without affecting the planet transits [23]. Here, we ignore other defects in the data, such as binary stars, sudden pixel-sensitivity dropouts, etc. Such data are, for example, collected by the Kepler Space Telescope [24] and the Transiting Exoplanet Survey Satellite (TESS) [25].

One would like to compare the hypothesis $H_{1}$ that we have a planet in the data to the null hypothesis that there is only noise. As argued in this paper, we will use the Bayes factor as a test statistic to incorporate informative prior information and non-Gaussian posteriors. The significance of a discovery is then reported as the probability of a false positive exceeding the observed value of the Bayes factor. We will first outline the procedure for calculating the Bayes factor given the prior for the transit model parameters. Then, using simulations of the null hypothesis, we will demonstrate that Equation (13) gives reliable results for the *p*-value of the Bayes factor (Section 4.3.2). In Section 4.3.3, we will discuss several prominent prior choices and demonstrate how a realistic prior choice can reduce the Type II error at a constant Type I error. In Section 4.4, we will consider a noise distribution with strong power-law tails and show that the *p*-value can still be computed by an analytical formula (11).

#### 4.3.1. Bayes Factor

The planet transit model m(t) can be parametrized by d=4 parameters: transit amplitude, period, phase, and transit duration, z=(A,P,ϕ,τ). The transit model is of the form

(22) $$m_{i}\left(z\right)=A\sum_{m=1}^{M}U\left(\frac{t_{i}-\left(m+\phi \right)P}{\tau }\right).$$

It is a periodic train of *M* transits with period *P*, phase ϕ, amplitude *A*, and transit duration τ. U(x) is a U-shaped transit template that is nonzero in the region (−1/2, 1/2) and depends on the limb darkening of the stellar surface [26].

The likelihood is Gaussian, and stationarity ensures that the Fourier transformation F diagonalizes the covariance matrix with the power spectrum on the diagonal. The power spectrum $P\left(\omega \right)=\langle |F\left\{n\right\}{|}^{2}\rangle$ can be learned from the data [27].

To make the noise standard Gaussian IID, we introduce

(23) $$x\overset{}{\to }\frac{F\{x\}}{P^{1/2}}\;m\overset{}{\to }\frac{F\{m\}}{P^{1/2}}.$$

We also rescale the amplitude *A* to make the model normalized.

It will be convenient to first optimize over the linear parameter $z_{1}$ and then over the remaining nonlinear parameters. The amplitude’s MAP value at fixed $z_{>1}$ is by Equation (A3)

(24) $${\hat{z}}_{1}\left(z_{>1}\right)=\frac{\int F{\left\{x\right\}}^{*}F\left\{m\right\}d\omega /P}{\sqrt{{\int |F\left\{m\right\}|}^{2}d\omega /P}},$$

where we have restored a model normalization factor. This is the matched filtering expression for the signal-to-noise ratio [23] and can be computed efficiently using Fourier transforms [23] for all phases ϕ on a fine grid at once. Our strategy for finding the highest MAP solutions is therefore to first find a maximum likelihood estimator (MLE) by scanning over the entire parameter space (for details, see [23]) and then use it as an initial guess in a nonlinear MAP optimization.

We calculate the posterior volume associated with the most promising peaks by marginalizing over the planet’s parameters in the vicinity of the peak. The amplitude has a Gaussian posterior which is not correlated with the other parameters, because the likelihood is Gaussian, and we are properly normalizing the template (see the discussion below Equation (A7)). The posterior volume is therefore

(25) $$V_{\mathrm{post}}=\sqrt{2\pi }\int \frac{p\left(z_{>1}\right)\;e^{{\hat{z}}_{1}{\left(z_{>1}\right)}^{2}/2}}{p\left(\hat{z}\right)\;e^{{\hat{z}}_{1}^{2}/2}}\;dz_{>1}.$$

**Figure 1:**
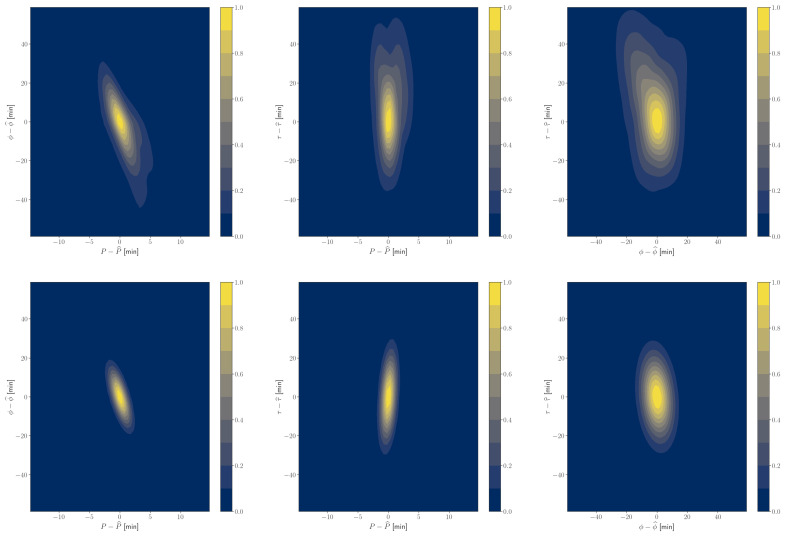
We show an example of a typical noise simulation with the injected planet with a period P=100 days and transit duration τ=0.4 days. We show the likelihood ratio in the neighborhood of the local MAP parameters $\hat{z}$ (upper panels). Laplace approximation expands the likelihood ratio’s logarithm to a quadratic order and approximates the peak with a Gaussian (lower panels). Note that the Laplace approximation is poor, corresponding to an inaccurate *p*-value estimation (Figure 2).

**Figure 2:**
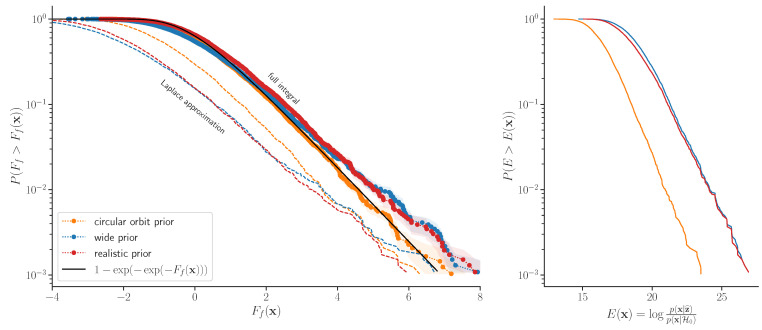
We simulate 4600 noise series and search for the planetary signal. In each realization, we find a candidate with highest Bayes factor and evaluate its *p*-value using Equations (11) and (13). In the left panel, we then compare it with a fraction of noise realizations where a more significant planet candidate was found. A good agreement between the empirical and analytical *p*-value is found, regardless of the prior choice. Dotted lines are the corresponding results using the asymptotic limit of Laplace approximation, which is inadequate in this case. The shaded regions are the bootstrap error estimates due to the finite number of simulations. They are a symmetric region around the mean value, which covers 68 out of 100 bootstrap draws. In the right panel, we show the MLR test statistic distribution for the null hypothesis using the same simulations. Note a large trials factor, which causes a noise-only simulation to produce an $\mathrm{SNR}=\sqrt{2E}>6$ in most realizations. There is an order-of-magnitude difference in the trials factor for the circular prior versus the wide or realistic prior.

One would be tempted to employ the Laplace approximation. Figure 1 and Figure 2 show this does not give satisfactory results: we are not in the asymptotic limit. While a frequentist approach using Wilks’ theorem would become invalid, here we can compute the full marginal integral of Bayesian evidence. Integral (25) is only three dimensional, so we take the Hessian at the peak to define the Gaussian quadrature integration scheme [28] of degree 7, implemented in [29], which requires 24 integrand evaluations.

#### 4.3.2. *p*-Value

To obtain the *p*-value, we use Equation (11) and extend it to all *p*-values using (13). We test our analytical expression for the *p*-value of the Bayes factor by simulating the null hypothesis and comparing the computed *p*-value with the empirically determined value. We evaluate 4600 realizations of the null hypothesis. We take a realistic power spectrum extracted from Kepler’s data for the star Kepler 90. The power spectrum and example realizations are shown in Figures 2 and 4 of [23]. A realization is a uniformly distributed choice of the phases of the Fourier components and normally distributed amplitudes of the Fourier components with a zero mean and variance, given by the power spectrum. In each of the resulting time series, we then determine the Bayes factor of the planet hypothesis against the null hypothesis (Section 4.3.1) and its *p*-value.

The analytic and empirical *p*-value are compared in Figure 2, achieving an excellent agreement. This shows that our formalism enables the evaluation of the *p*-value beyond the asymptotic limit, thus generalizing Wilks’ theorem.

#### 4.3.3. The Choice of Prior

Here, we discuss a prior choice for the period, phase, and transit duration. We will see that using an informative prior when available can significantly improve detection efficiency.

We scan over the period range from 3 to 300 days, and at each period over all phases from 0 to 1. As we argued in this paper, when we do not know the prior, it is simplest to adopt Jeffrey’s choice. We will assume this for the period and phase parameters. Note that for the phase, Jeffrey’s prior is flat from 0 to 1, which we can also view as a known (informative) prior because the phase cannot affect the exoplanet detectability. There is a natural value for the transit duration parameter τ, given the planet’s period and assuming the circular, non-inclined orbit. We illustrate the impact of the prior choice by considering three scenarios:

(1) A fixed value of τ, defined by assuming a circular, perfectly aligned orbit. The transit duration is fixed by the Kepler’s third law: $\tau_{K}\left(P\right)={\left(3P/\left(\pi^{2}G\rho_{*}\right)\right)}^{1/3}$, where $\rho_{*}$ is the star’s density. This choice can be too restrictive for non-circular or inclined orbits and can penalize real planets.
(2) Jeffrey’s prior on a broad domain $\tau \in [\tau_{K}\left(P_{min}\right),\;2\;\tau_{K}\left(P_{max}\right)]$. This choice may include physically implausible transit times, leading to a larger multiplicity penalty.
(3) A realistic prior distribution, taking into account inclined and eccentric orbits. Orbits are assumed to be isotropic, with eccentricities drawn from a beta function with parameters that match the observed planets in the Kepler’s data [30]. In addition, the star’s density is measured with an uncertainty on the order of 15%, causing uncertainty in $\tau_{K}$. The transit duration prior is obtained by marginalizing over the orbit inclination, eccentricity, and star’s parameters. The distribution is a broadened version of the delta function at $\tau_{K}$.

**Figure 3:**
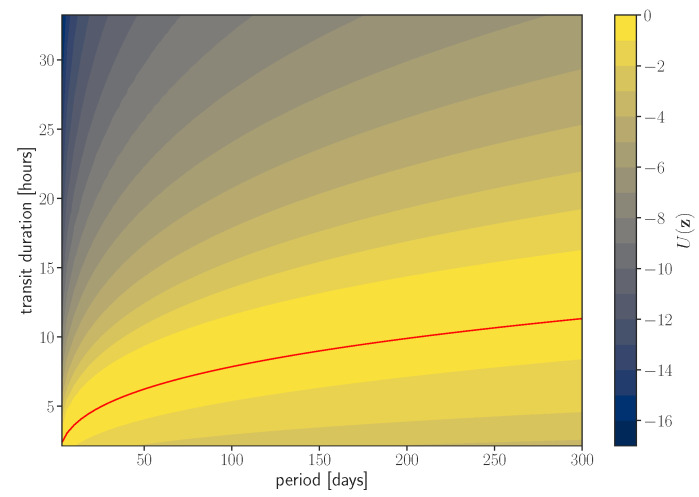
We show the potential $U=\log p\left(z\right)/p_{Jeff}\left(z\right)$ for different choices of the τ prior. This quantity measures to what extent is the prior informative, with the Jeffrey’s prior being non-informative. This potential directly impacts the hypothesis test’s outcome, see Section 5. Fixing τ to the Kepler value is equivalent to a constant potential on the red line and −∞ everywhere else. A broad Jeffrey’s prior is equivalent to a constant potential over the entire search domain. The informative prior is shown with the color scale. It peaks close to the Kepler’s value but is broadened to account for variation in planet eccentricity, orbit orientation, and stellar density uncertainty.

**Figure 4:**
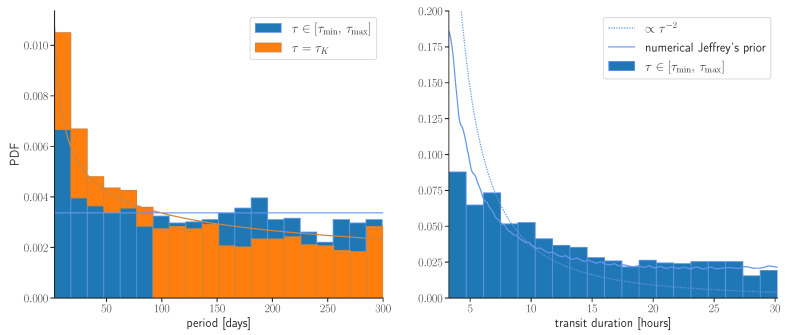
We show marginal probability density distributions over the period and transit duration of the five highest $F_{f}$ candidates from each of the 300 noise-only simulations. The distributions follow generalized Jeffrey’s prior, which is approximated reasonably well with the scaling estimates from Equations (A18) and (A19). This confirms that regions with smaller $V_{\mathrm{post}}$ have more false positives. The blue plot is showing a situation where the transit duration is a free parameter in the search, yielding p(P)∼const and $p\left(\tau \right)∼\tau^{-2}$. A simple analytical estimate does not give an accurate Jeffrey’s prior for the transit duration because of the non-white power spectrum. Therefore, we complement this analysis with a numeric evaluation of the expected posterior volume (solid line) and show that this gives a better match. The orange plot shows a situation where the transit duration is fixed to the Kepler’s value. It is well described by the scaling estimate $p\left(P\right)\propto P^{-1/3}$.

All three choices are visualized in Figure 3. In Appendix C, we derive the approximate Jeffrey’s prior by deriving the scaling of the Fisher information matrix. In the first two scenarios, we, respectively, obtain $p\propto P^{-1/3}$ and $p\propto \tau^{-2}$. One can take the transit template and calculate the Fisher information matrix numerically for more accurate results. In Figure 4, we show that the peaks of the null hypothesis are distributed by Jeffrey’s prior, in agreement with our predictions.

Taking τ as a completely unconstrained parameter will in general lead to detecting planets that are not physically plausible and will therefore increase the false-positive rate and force us to reject more real planets than necessary. In the present example, shown in Figure 2, this effect is not very large if the prior is chosen such that it still introduces a reasonable cutoff on the transit duration. However, choosing such a cutoff is a choice that must be based on physical arguments: the prior plays an essential role.

A prior that is too narrow (case 1) is also suboptimal because it will reject some real planets. Using a template with duration $\tau_{K}$ when in fact a planet has transit duration $\tau \neq \tau_{K}$ will reduce the planet’s SNR by

(26) $$\frac{\mathrm{SNR}(\mathrm{detected})}{\mathrm{SNR}(\mathrm{true})}=\frac{\int F{\left\{m_{\tau }\right\}}^{*}F\left\{m_{\tau_{K}}\right\}d\omega /P}{\int F{\left\{m_{\tau_{K}}\right\}}^{*}F\left\{m_{\tau_{K}}\right\}d\omega /P},$$

where this is an expected reduction over the noise realizations.

**Figure 5:**
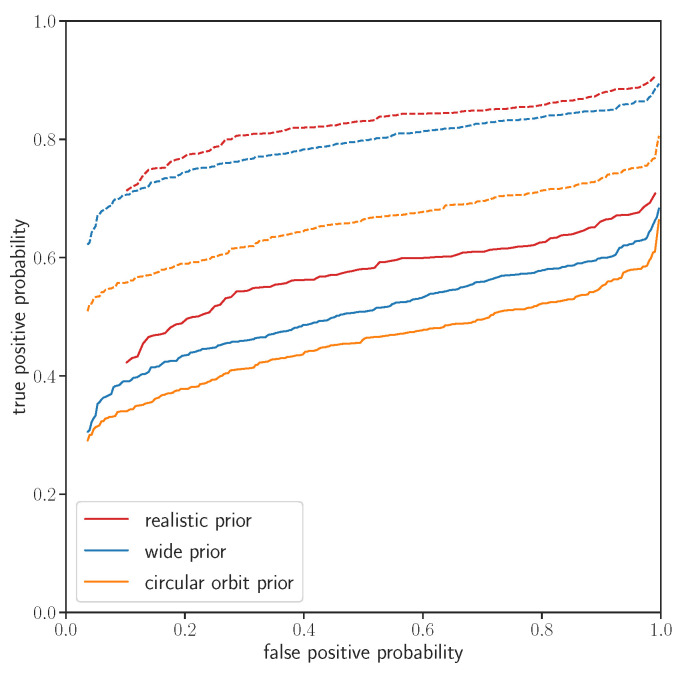
ROC curve (see Section 4.3.3) defined as true-positive probability, i.e., power (1-Type II error) versus false-positive probability, i.e., Type I error (*p*-value), for various prior choices. We search for a hypothetical small planet with injected radius $R=1.45R_{⨁}$, corresponding to SNR(true)=6.25 (solid lines), and $R=1.8R_{⨁}$ and SNR(true)=7 (dotted lines). Using the realistic prior results in an improved ROC curve (higher true-positive probability at a given false-positive probability), implying that the Bayes factor with a realistic prior is the test statistic with the highest statistical power. The true-positive probability at a fixed *p*-value is decreased for the circular orbit prior because the prior does not include all the allowed signal template forms. On the other hand, a wide prior includes too many signal template forms that include signal templates that occur rarely or never, leading to a larger multiplicity and a larger false-positive rate at a constant true-positive probability.

In Figure 5, we show the ROC curves (Receiver-Operated Characteristic) for all three prior choices, that is, a true-positive rate as a function of the false-positive rate, both parametrized by the detection threshold. The false-positive probabilities are taken from Figure 2. The true-positive probability is a probability that the signal with some SNR(true) is detected above the threshold. The detected SNR can be approximated as a Gaussian-distributed variable with unit variance. Its mean is μ=SNR(true) with the realistic and wide priors. It is additionally reduced with the circular orbit prior because the search template differs from the true template (Equation (26)). Using a realistic prior improves the true detection probability at a fixed false-positive probability relative to a prior that is too narrow (a circular orbit prior) because it improves the fit. It also improves the ROC relative to a prior that is too broad because it does not include the templates that rarely happen in the data and would lead to a larger multiplicity and hence a larger false-positive rate. This figure thus demonstrates that hypothesis testing with a realistic prior Bayes factor gives an optimal ROC (power versus *p*-value).

### 4.4. Non-Gaussian Likelihood

Our final example is a single exoplanet transit search in the Student’s t-distributed noise:

(27) $$q\left(x_{i}\right)\propto {\left(1+x_{i}^{2}/\nu \right)}^{-(\nu +1)/2},$$

where ν is a parameter of the distribution (degrees of freedom). $\nu \overset{}{\to }\infty$ corresponds to the Gaussian noise, while lower values of ν result in strong power-law tails. This noise is an exaggerated version of the outlier distribution in the actual Kepler data [27]. The alternative model is a single U-shaped transit with a fixed transit duration τ=1.2 days. The parameters of the model are the location of the transit ϕ and its amplitude *A*.

**Figure 6:**
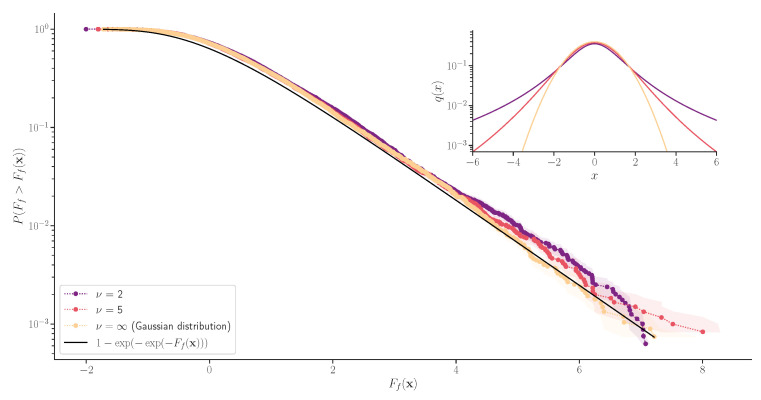
False-positive probability of a search for a single exoplanet transit in the power-law outlier noise. We consider Student’s t-noise distributions with ν = 2, 5, and *∞*. The noise probability density functions of the data are shown in the inset figure. In each case, we simulate 7000 null data realizations and plot the distribution of the $F_{f}$-test statistic. We have an excellent agreement with the *p*-value prediction of Equation (13) in black.

As in the main exoplanet example, we have a time series of flux measurements, equally spaced every 30 min, and the total length here is 200 days. We do not consider time-domain noise correlations here. Matched filtering is not possible due to the non-Gaussian noise, so we scan over the phase in 30 min increments and find the MLE $z_{1}$ solution at each phase using the Newton’s method. We identify the five highest MLR peaks and compute the Bayes factor around each peak. We find that the Laplace approximation is accurate for the amplitude parameter, but the ϕ integral has to be taken numerically. We take the peak with the highest Bayes factor, compute the associated $F_{f}$ of Equation (11), and see an excellent agreement with the *p*-value prediction (13), shown in Figure 6.

## 5. Statistical Mechanics Interpretation

Here, we interpret the Bayesian and the frequentist hypothesis testing as an energy versus entropy competition, where the energy is the maximum log-likelihood which favors the alternative model and the entropy is the influence of the look-elsewhere effect.

In a continuous parameter space, the nearby models are not independent. In fact, we can consider the models which cannot be distinguished after seeing the posterior as one independent unit, which we call a state. This is analogous to the shift from classical statistical mechanics to quantum statistical mechanics, where the discrete states are counted in units of their uncertainty volume. Similar ideas were used in [13]. In this context, the generalized Jeffrey’s prior is non-informative in the sense that it assigns an equal probability to each state [13], so to each effective indistinguishable model.

### 5.1. Bayes Factor

Using the Jeffrey’s prior, the logarithm of the Bayes factor (3)

(28) $$F\equiv \log B=E-\log\frac{1}{p\left(\hat{z}\right)V_{\mathrm{post}}\left(\hat{z}\right)}=E-\log N\equiv E-S$$

is reminiscent of the thermodynamics relation for the free energy. We identify *E* with energy and S=logN with entropy. Note that *N* is independent of $\hat{z}$ and equals the total number of states that fit in the prior volume [31]).

For a general prior, the thermodynamics relation has to be generalized to F=E+U−S, where the potential energy $U=\log\frac{p(z)}{p_{Jeff}\left(z\right)}$ measures the extent to which the prior is informative, meaning that it favors some states over the others.

Note that the entropy is always positive and therefore always favors $H_{0}$, because the posterior is narrower than the prior. The energy has to surpass the entropy for the alternative hypothesis to prevail. This is the Occam’s razor penalty, which is built into the Bayes factor.

Our definitions of energy and entropy should be viewed from the hypothesis testing point of view, where the energy E+U is the only “macroscopic” parameter that influences the outcome of the test. The other parameters are “microscopic” in the sense that we do not care about their values in the test. Entropy is the logarithm of the number of microstates, given the macrostate, as usual. To be precise, the entropy should only count the states which give rise to the same macrostate, so the states with the same energy. Such a count corresponds to the Bayes factor, which ignores the look-elsewhere effect associated with the amplitude parameter. This makes little sense from the Bayesian perspective as the prior is determined only after seeing the data, such that the amplitude is fixed to its MAP value under the original prior. However, we have seen that it is exactly the reduced Bayes factor $B_{>1}$ from Equation (10) that appears in the frequentist analysis.

### 5.2. *p*-Value

We recognize that the integral $\int \frac{dz}{V_{\mathrm{post}}\left(z\right)}$ in Equation (9) is the continuous version of the sum over states. The asymptotic *p*-value $P_{\mathrm{asym}}$ is therefore the sum over all states which generate false positives, each weighted with the Boltzmann factor $e^{-E}$:

(29) $$P_{\mathrm{asym}}\left(F>F\left(x\right)\right)=\sum_{F(\mathrm{state})>F(x)}e^{-E(\mathrm{state})}=e^{-F_{f}}.$$

However, this is exactly the partition function of the canonical ensemble with $k_{B}T=1$, so $F_{f}$ is the frequentist definition of the free energy. The *p*-value approximation (11) implies that the Bayesian free energy $\log B_{>1}$ and the frequentist free energy $F_{f}$ are one and the same thing, up to the logarithmic corrections in the energy. Note that in physics, the thermodynamic and statistical mechanic free energies are also the same in the thermodynamic limit.

**Figure 7:**
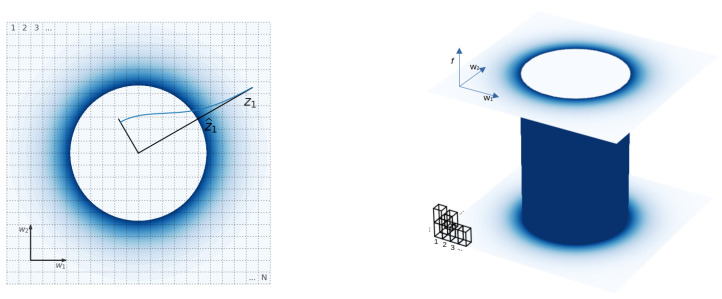
Illustration of the statistical mechanics interpretation of the *p*-value. We emphasize the difference between the amplitude and the other parameters. Left panel: Parameter space of a linear model (15) with two degrees of freedom. This is equivalent to the periodogram model (18) at a fixed frequency ω. Constant-energy (MLR) shells are circles of radius $z_{1}={\left(w_{1}^{2}+w_{2}^{2}\right)}^{1/2}$. We assume a uniform prior with equal uncertainty on both variables and show posterior volume as the area of each mesh cell. The expected ratio of the null hypothesis to the alternative hypothesis events in each cell is given by the Boltzmann factor in Equation (29). It dies off exponentially as a function of energy. The color intensity in the plot is proportional to this Boltzmann factor, which is shown along one radial direction as a blue line. False-positive rate counts all the states in the region exterior to the circle of observed F(x) and weights them with the Boltzmann factor (29). Only the region close to the observed test statistic circle will contribute due to the exponential suppression. Increasing the prior on $w_{1}$ and $w_{2}$ increases the total number of states and reduces the Bayes factor but has no impact on the *p*-value (17). Right panel: Now, we also vary the frequency, searching for sinusoidal signal (18) over all frequencies within some prior range. The observed F(x) surface is a cylinder, and the false-positive rate is proportional to its surface area. Increasing the prior range of ω over which we search for sinusoidal signal reduces the Bayes factor because additional states were introduced (16). Contrary to the first example, the false-positive rate has now also increased (21) because some new states are close to the observed F(x) shell.

Note that in the frequentist analysis, not all the states contribute to the look-elsewhere effect equally but according to their Boltzmann factor. In other words, trials with parameters which are very unlikely under the null hypothesis do not increase the multiplicity. We show an illustration of this phenomenon in Figure 7.

## 6. Discussion

This paper compares the frequentist and Bayesian significance testing between hypotheses of different dimensionality, where the null hypothesis is a well-defined accepted model for the reality of the data, while the alternative hypothesis tries to replace the null hypothesis. Both the Bayesian and frequentist methodologies have advantages and disadvantages in the setting where the alternative hypothesis has not been observed with sufficient frequency to develop a reliable prior. We argue that for optimal significance testing, the Bayes factor between the two hypotheses should be used as the test statistic with the highest power. However, the Bayes factor should not be used to quantify the test significance when the prior of the alternative hypothesis is poorly known. Instead, the frequentist false-positive rate of a null hypothesis (Type I error or *p*-value) can be used, which only depends on the properties of the null hypothesis, which is assumed to be well understood. The sensitivity of the Bayes factor to the choice of prior is known in the context of Lindley’s paradox [32]. While there is no actual paradox, it highlights the dependence of the Bayes factor on the choice of prior, which is undesirable because we often do not know it. Our solution to this paradox is to relate the Bayes factor to the *p*-value, which is independent of the alternative hypothesis, as it only tests the distribution of the null hypothesis. In this way, one can use the *p*-value for hypothesis testing even when using Bayesian methods, by using the Bayes factor as a test statistic.

While it is common to use the MLR as a test statistic, this is not prior independent but corresponds to the generalized Jeffrey’s prior. If some prior information is available, as in our exoplanet example of transit duration being determined by the period *P* via the Kepler law, one should use it to reduce the Type II error. We note that Jeffrey’s prior can be unreasonable, even in unknown prior situations: if, in a given experiment, the posterior volume is strongly varying across the parameter space, the Jeffrey’s prior is very experiment-specific. A prior that is smooth across the parameter space is undoubtedly a better prior, even if we do not know what the specific form should be. Nevertheless, when Jeffrey’s prior is reasonable, it simplifies the analytic calculation of the *p*-value.

We show that both the *p*-value and the Bayes factor can be expressed as an energy versus entropy competition. We define energy as the maximum logarithm of the likelihood ratio and Bayesian entropy as the logarithm of the number of posterior volumes that fit in the prior volume. The constant-energy Bayes factor is analogous to the thermodynamical free energy. Conversely, the *p*-value in the asymptotic regime corresponds to the canonical partition function, which is a Boltzmann factor weighted sum over the posterior states with the test statistic above the observed one. In the low *p*-value regime, only the states close to the observed test statistic contribute to the sum. Therefore, the constant-energy Bayes factor and the asymptotic *p*-value are related. This also happens in physics, where the statistical and thermodynamical definitions of the free energy coincide in the thermodynamical limit. As an example, we show that the *p*-value of the standard $\chi^{2}$ distribution of *d* degrees of freedom can be interpreted as an energy versus entropy competition, with the latter defined as the logarithm of the number of states on the constant-energy shell. The entropy grows as the log of the area of a sphere in *d* dimensions with a radius proportional to the square root of the energy.

The formalism developed here extends the Wilks’ theorem [15] in several ways. First, the connection to the Bayes factor allows us to define the posterior volume beyond the Gaussian approximation inherent in the asymptotic limit assumed for the Wilks’ theorem. Second, the Wilks’ theorem assumes the parameter values are inside the boundaries. We show that a generalization counting the states as a function of energy provides a proper generalization that gives better results. Third, Wilks’ theorem does not account for the multiplicity of the look-elsewhere effect. Our method correctly handles these situations.

As an example application, we apply the formalism to the exoplanet transit search in the stellar variability-polluted data. We search for exoplanets by scanning over the period, phase, and transit duration, and we show that the multiplicity from the look-elsewhere effect is of order $10^{7}$. We find that the Laplace approximation for the uncertainty volume $V_{\mathrm{post}}$ is inaccurate, while the numerical integration of the Bayesian evidence gives very accurate results when compared to simulations. We emphasize the role of informative priors, such as the planet transit time prior, which reduce the Type II error, leading to a higher fraction of true planets discovered at a fixed Type I error (*p*-value) threshold. The method enables a fast evaluation of the false-positive rate for every exoplanet candidate without running expensive simulations.

There are other practical implications that follow from our analysis. For example, the multiplicity depends not only on the prior range but also on the posterior error on the scanning parameters. If this error is small in one part of the parameter space but large in others, then the MLR test statistic leads to a large multiplicity that will increase the *p*-value for all events. We show that analytic predictions reproduce the distribution of false positives as a function of the period and transit duration. An informed choice of the prior guided by what we know about the problem and what our goals are may change this balance and reduce the multiplicity penalty, thus reducing the Type II error: the Bayes factor can be a better test statistic for the Type II error than the MLR. One is of course not allowed to pick and choose the prior a posteriori: we must choose it prior to the data analysis.

In many situations, it is possible to analytically obtain the false-positive rate as a function of the Bayes factor test statistic from Equation (11), which gives a *p*-value estimate that is more reliable for hypothesis testing than the corresponding Bayes factor in situations of a new discovery where the prior is not yet known. As a general recommendation, we thus advocate that Bayesian analyses report the frequentist *p*-value using the Bayes factor test statistic against the null hypothesis as a way to quantify the significance of a new discovery, and that frequentist analyses use the Bayes factor as the optimal test statistic for hypothesis testing while using frequentist methods to quantify its significance.

## Figures and Tables

**Figure A1:**
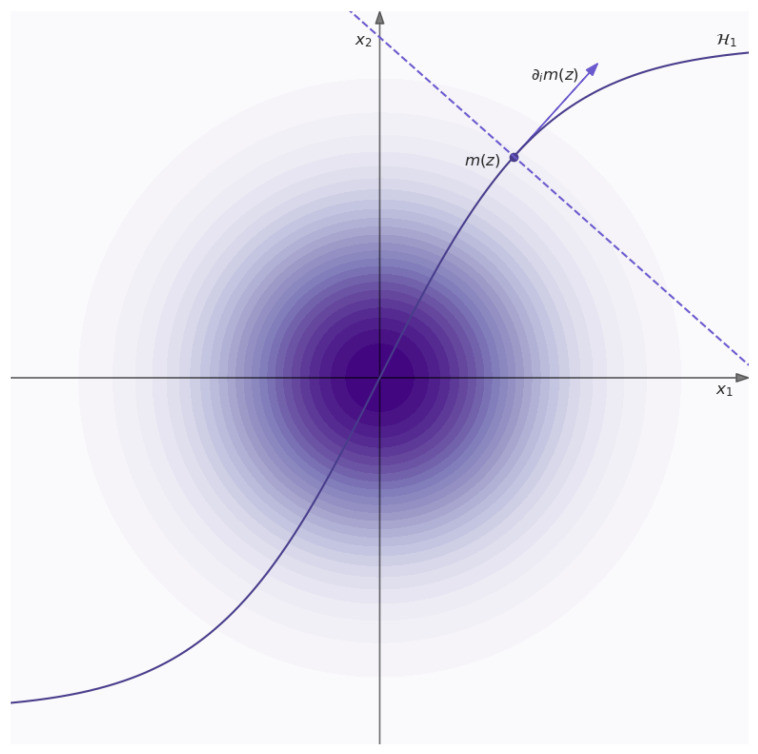
Illustration of a two-dimensional data space (n=2). The concentric circles are the likelihood contours of $H_{0}$. The solid line is the $H_{1}$ manifold (d=1). If the data point x lies on the dashed line, the model m(z) satisfies Equation (A3) and is at least locally the MLE model. The integral of Equation (A10) runs over the dashed line.

## Data Availability

The data products associated with the Kepler data analysis in Section 4.3 are available in the NASA Exoplanet Archive, at https://exoplanetarchive.ipac.caltech.edu/bulk_data_download/ (accessed on 18 September 2022). Other data associated with this article will be shared upon reasonable request to the corresponding author.

## Abbreviations

The following abbreviations are used in this manuscript:

FPR	False-Positive Rate
IID	Independently Identically Distributed
MAP	Maximum a Posteriori
MLE	Maximum Likelihood Estimator
MLR	Maximum Likelihood Ratio
ROC	Receiver-Operated Characteristic
SNR	Signal-to-Noise Ratio

## Appendix A. Gaussian Likelihood

We will here assume the likelihood is Gaussian and the hypotheses only differ in their prediction for the data mean. This is a common situation that applies in our examples in Section 4.1, Section 4.2 and Section 4.3. We will better motivate some of the assumptions that we needed to derive our *p*-value expressions (9) and (11). Furthermore, we will present a complementary geometric approach and re-derive the *p*-value expressions from the frequentist perspective, so without using the concept of a prior.

### Appendix A.1. Laplace Approximation for the Amplitude Is Exact

Because the null hypothesis has no free parameters, we can choose to work in the coordinates in which the likelihood covariance matrix is the identity matrix and the mean prediction under the null hypothesis is zero. The negative log-likelihood of the data is then

(A1) $$\begin{array}{cc} -\log p(x|H_{0}) & =\frac{1}{2}{∥x∥}^{2}\\ -\log p(x|H_{1}) & =\frac{1}{2}{∥x-m\left(z\right)∥}^{2}, \end{array}$$

where z is the parameters of the alternative hypothesis. Let us decompose the alternative model as

(A2) $$m\left(z\right)=z_{1}M\left(z_{>1}\right),$$

where $z_{1}$ is the amplitude and $M(z_{>1})$ is the template, which depends on the nonlinear parameters $z_{>1}=\left(z_{2},\;z_{2},\;...,\;z_{d}\right)$. By suitably rescaling $z_{1}$, we can take the template to be normalized, $∥M\left(z_{>1}\right){∥}^{2}=1$, where the norm is the $l^{2}$ norm.

We will assume that the prior variations are much slower than the likelihood variations, so the MAP model $m(\hat{z})$ is the point on the model manifold, which is locally the closest to the data. It has to satisfy:

(A3) $$\left(x-m\right)\cdot \partial_{i}m=0\;i=1,\;2,\;...,\;d.$$

The Gaussian MLR is directly related to ${\hat{z}}_{1}$:

(A4) $$\begin{array}{cc} E & \equiv \log\frac{p(x|\hat{z})}{p(x|H_{0})}=-\frac{1}{2}\left({∥x∥}^{2}-{∥x-m\left(\hat{z}\right)∥}^{2}\right)=\frac{1}{2}\left(2x\cdot m\left(\hat{z}\right)-{∥m\left(\hat{z}\right)∥}^{2}\right)=\frac{1}{2}{\hat{z}}_{1}^{2}. \end{array}$$

In the last step, we have used $x\cdot m\left(\hat{z}\right)=m\left(\hat{z}\right)\cdot m\left(\hat{z}\right)$ by Equation (A3) for i=1. Note also that $∥m\left(\hat{z}\right){∥}^{2}={\hat{z}}_{1}^{2}$ by Equation (A2) and the choice of normalization. We can therefore identify ${\hat{z}}_{1}$ as the signal-to-noise ratio.

### Appendix A.2. Posterior Volume Approximately Depends on the Local MAP Parameters Only

The posterior is in general not Gaussian, and the Laplace approximation may be a poor approximation (e.g., Section 4.3), but we first adopt it anyway to gain some intuition. Equation (4) gives the posterior covariance matrix:

(A5) $$\Sigma_{ij}^{-1}=\partial_{i}m\cdot \partial_{j}m-\left(x-m\right)\cdot \partial_{i}\partial_{j}m.$$

The first term is independent of the data and equals the Fisher information matrix (5)

(A6) $$I_{ij}=\partial_{i}m\cdot \partial_{j}m,$$

while we drop the second term because x−m is rapidly oscillating around zero if there are no systematic residuals and the Hessian of the model is typically varying more slowly (Gauss–Newton approximation).

Although the Bayes factor (3) is in general a function of the data x, we have here approximated it solely as a function of the best fit parameters $\hat{z}$, inferred from the data:

(A7) $$B\left(\hat{z}\right)=\frac{{\left(2\pi \right)}^{d/2}p\left(\hat{z}\right)}{{\left[\det I\left(\hat{z}\right)\right]}^{1/2}}\exp\{\frac{1}{2}{\hat{z}}_{1}^{2}\}.$$

Note that the Fisher determinant can be restricted to the $z_{>1}$ components only, because there are no correlations between $z_{1}$ and the other parameters: $I_{1i}=M\cdot z_{1}\partial_{i}M=\frac{1}{2}z_{1}\partial_{i}{∥M∥}^{2}=0$ and $I_{11}={∥M∥}^{2}=1$. This property is exact and does not rely on the Laplace approximation, as can be seen by using the Gaussian likelihood (A1) in the Bayes factor computation (1). We also note that the errors on the nonlinear parameters scale as the inverse signal-to-noise ratio, because $I_{ij}=z_{1}^{2}\;\partial_{i}M\cdot \partial_{j}M$, but this is only true under the Fisher approximation to the posterior.

Using the Gauss–Newton approximation in the Laplace approximation, we were able to show that the Bayes factor depends on the data only through the MAP parameters, inferred from the data, but not on the specific data realization. This is true even if the Laplace approximation fails. The posterior volume is then

(A8) $$\begin{array}{cc} \; & V_{\mathrm{post}}\left(x\right)=\int \frac{p(x|z)p(z)}{p\left(x|\hat{z}\right)p\left(\hat{z}\right)}dz=\int \frac{p(z)}{p(\hat{z})}\;e^{-\frac{1}{2}{(∥x-m\left(z\right)∥}^{2}-∥x-m\left(\hat{z}\right){∥}^{2})}dz\\ \; & =\int \frac{p(z)}{p(\hat{z})}\;e^{-\frac{1}{2}{∥m\left(z\right)-m\left(\hat{z}\right)∥}^{2}}e^{-\left(x-m\left(\hat{z}\right)\right)\cdot \left(m\left(\hat{z}\right)-m\left(z\right)\right)}dz\approx \int \frac{p(z)}{p(\hat{z})}\;e^{-\frac{1}{2}{∥m\left(z\right)-m\left(\hat{z}\right)∥}^{2}}dz=V_{\mathrm{post}}\left(\hat{z}\right), \end{array}$$

where in the approximation we have used $m(\hat{z})$ as the stationary model of the likelihood (see Equation (A3)) if the prior is sufficiently smooth; therefore, the exponent in the second factor vanishes at the linear order in $z-\hat{z}$.

### Appendix A.3. Frequentist Derivation of p-Value

We will here present an approach that is complementary to Section 3 in the sense that it does not use any of the Bayesian concepts but still reproduces the same results. Because there is no concept of a prior, we will be working with the MLR as a test statistic (which is approximately the Bayes factor with the generalized Jeffrey’s prior). We will assume the setup as in Appendix A.

First, let us determine how likely we would have seen some model m(z) as the MLE model if the null hypothesis was true. We add the probabilities over the suitable region of the data space:

(A9) $$p\left(m\left(z\right)|H_{0}\right)=\int_{N}p\left(x|H_{0}\right)dx,$$

where N is the set of all points which have m(z) as their MLE model. N is a subset of the plane defined by Equation (A3). This equation defines an affine n−d-dimensional plane which is an orthogonal complement to the tangent plane to the $H_{1}$ manifold at the point m(z) (for illustration, see Figure A1). However, N is not the entire plane because Equation (A3) is a necessary but not sufficient condition. In other words, the posterior may be multi-modal. To make progress, we will have to neglect this and assume N to be the entire plane. By doing so, our final result for the *p*-value is in fact an upper bound, which becomes more and more accurate the lower the *p*-value.

The model m(z) is both a point on the plane N and a normal to the plane, as can be seen from Equation (A3) with i=1. Writing $x=m\left(z\right)+x_{⊥}$ for points on the plane and using orthogonality, we can easily evaluate the Gaussian integrals (A9):

(A10) $$p\left(m\left(z\right)|H_{0}\right)=\int p\left(x|H_{0}\right)dx_{⊥}=\frac{e^{-{∥m∥}^{2}}/2}{{\left(2\pi \right)}^{d/2}}.$$

This result is saying that the probability density in the data space of a model occurring as an MLE model under $H_{0}$ depends only on $\frac{1}{2}{∥m∥}^{2}=E$. Therefore, the probability of observing *E* under the null hypothesis equals the probability of finding any m with $\frac{1}{2}{∥m∥}^{2}=E$ as the MLE model. This gives

(A11) $$p\left(E|H_{0}\right)=\frac{1}{\sqrt{2E}}\frac{e^{-E}}{{\left(2\pi \right)}^{d/2}}V_{\mathrm{shell}},$$

where $V_{\mathrm{shell}}$ is the data-space volume of models with $\frac{1}{2}{∥m∥}^{2}=E$, in other words, the constant MLR surface volume. We have picked up a factor ${\left(2E\right)}^{-1/2}$ when transforming the density with respect to ${\hat{z}}_{1}$ to the density with respect to *E*. The volume of the constant MLR shell in the data space is an integral over the $H_{1}$ manifold at fixed $z_{1}=\sqrt{2E}$. It can be evaluated in the $z_{>1}$ parameter space by integrating the square root of the determinant of the metric over the coordinate range:

(A12) $$V_{\mathrm{shell}}=\int \;dm\left(\sqrt{2E},z_{>1}\right)=\int \det I_{ij}{\left(\sqrt{2E},z_{>1}\right)}^{1/2}dz_{>1},$$

where the metric on the $H_{1}$ manifold coincides with the Fisher information matrix (A6). Combining Equations (A11) and (A12), we find

(A13) $$p\left(E|H_{0}\right)=\frac{e^{-E}}{\sqrt{2E}}\int \frac{dz_{>1}}{V_{\mathrm{post}}\left(\sqrt{2E},z_{>1}\right)},$$

where $V_{\mathrm{post}}={\left(2\pi \right)}^{d/2}\det I_{ij}^{-1/2}$.

Even though we use frequentist formalism, the expected posterior volume enters as the determinant of the transformation from the data space to the parameter space. We reproduce the result of Equation (9).

There are some exciting applications where the derived expressions are exact (see Section 4.1) because the posterior has a single mode. However, in general, the frequentist derivation made it manifest that in the presence of multiple modes, the *p*-value results are only an upper bound, which becomes useless if the *p*-value is high. In that case, one can apply a correction (13) if the posterior is narrow compared to the prior.

## Appendix B. Fisher Analysis of the Periodogram

We here perform the Fisher analysis of the periodogram example in Section 4.2 by employing Equation (A7). To make the calculations tractable, we assume dense data sampling over many periods of the signal ($t_{i+1}-t_{i}\ll 1/\omega \ll T$) so that we can replace the discrete sum in the scalar products of Appendix A with the integral $\sum_{i=1}^{n}\overset{}{\to }\frac{n}{T}\int_{0}^{T}dt$, where *T* is the total time of the measurements. We will work with the amplitude $z_{1}$ relative to the normalized template M, which has A=. The log-MLR is $E=\frac{1}{2}{\hat{z}}_{1}^{2}$ by Equation (A4).

We compute the Fisher matrix (A6) component by component, for example,

(A14) $$\begin{array}{cc} \; & I_{\phi \omega }={\hat{z}}_{1}^{2}\;\partial_{\phi }M\cdot \partial_{\omega }M=\frac{{\hat{z}}_{1}^{2}}{\sigma^{2}}\sum_{k}\;\partial_{\phi }M_{k}\partial_{\omega }M_{k}\\ \; & \approx \frac{{\hat{z}}_{1}^{2}}{\sigma^{2}}\frac{n}{T}\int_{0}^{T}\sigma \sqrt{\frac{2}{n}}\partial_{\phi }\sin\left\{\omega t+\phi \right\}\sqrt{\frac{2}{n}}\partial_{\phi }\sin\left\{\omega t+\phi \right\}dt\\ \; & =\frac{4E}{T}\int_{0}^{T}\cos^{2}\left\{\omega t_{\phi }\right\}tdt\underset{T\gg 1/\omega }{\approx }\frac{4E}{T}\frac{1}{2}\int_{0}^{T}tdt=TE, \end{array}$$

and take the inverse to obtain

(A15) $$\left[\begin{array}{cc} \sigma_{\omega }^{2} & \sigma_{\omega \phi }\\ \sigma_{\omega \phi } & \sigma_{\phi }^{2} \end{array}\right]\equiv I^{-1}={\left[\begin{array}{cc} 2T^{2}E/3 & TE\\ TE & 2E \end{array}\right]}^{-1}=E^{-1}\left[\begin{array}{cc} 6/T^{2} & -3/T\\ -3/T & 2 \end{array}\right].$$

Interestingly, the frequency-phase correlation is relatively high $\sigma_{\omega \phi }/\sigma_{\omega }\sigma_{\phi }=-\sqrt{3}/2\approx -0.87$.

## Appendix C. Jeffrey’s Prior in the Exoplanet Example

Here, we derive the approximate Jeffrey’s prior in the exoplanet example by analyzing the Fisher information matrix (A6):

(A16) $$I_{ij}\left(z\right)=Re\int \frac{\partial F\{m\}}{\partial z_{i}}\frac{\partial F{\left\{m\right\}}^{*}}{\partial z_{j}}\frac{d\omega }{P(\omega )}.$$

Let us first determine the scaling of the $I_{\phi \phi }$. Fourier transforming the signal from Equation (22), differentiating it with respect to ϕ, inserting into Equation (A16), and expressing the amplitude $A=z_{1}$ with *E*, we obtain

(A17) $$I_{\phi \phi }=2E\frac{\int \frac{\omega^{2}P^{2}{\left|F\left\{m\right\}\right|}^{2}d\omega }{P(\omega )}}{\int \frac{{\left|F\left\{m\right\}\right|}^{2}d\omega }{P(\omega )}}\propto \frac{P^{2}}{\tau^{2}}\;,$$

where in the second step we change the variable of integration to the dimensionless quantity ωτ and neglect a residual mild dependence of the dimensionless integral on *P* and τ.

In a scenario where τ is an independent quantity, we similarly obtain $I_{PP}\propto P^{-2}\tau^{-1}$ and $I_{\tau \tau }\propto \tau^{-1}$; therefore, the Jeffrey’s prior is:

(A18) $$p\left(z\right)\propto \det I^{1/2}\propto \tau^{-2}\;.$$

In the other scenario, where τ is fixed by the period, we have $I_{PP}\propto P^{-2}$ and Jeffrey’s prior is:

(A19) $$p\left(z\right)\propto \tau_{K}^{-1}\propto P^{-1/3}\;.$$

An estimate of the Jeffrey’s prior is obtained by normalizing in the given range of the parameters.
